# The principle of uncertainty in biology: Will machine learning/artificial intelligence lead to the end of mechanistic studies?

**DOI:** 10.1371/journal.pbio.3002495

**Published:** 2024-02-08

**Authors:** Victor de Lorenzo

**Affiliations:** Systems Biology Department, Centro Nacional de Biotecnología, CSIC, C/ Darwin, Madrid, Spain

## Abstract

Molecular Biology has long tried to discover mechanisms, considering that unless we understand the principles, we cannot develop applications. Now machine learning and artificial intelligence enable direct leaps to application without understanding the principles. Will this herald a decline in mechanistic studies?

Biology as a scientific and research domain seems to have undergone major breakthroughs and paradigm shifts every time external disciplines have intersected with it. Typically, after a given time of somewhat uneasy coexistence, the community embraces the new conceptual frame and its associated technologies as a lens through which biological phenomena can be (re)interpreted. The happy encounter between biology and chemistry gave birth to biochemistry, enzymology, and metabolism. Much later, the interest of post-war physicists for live systems brought about the onset of molecular biology, which reached its biggest milestones in the elucidation of the DNA double helix and the deciphering of the genetic code. During the many decades dominated by molecular biology and molecular genetics, the emphasis has been on mechanistic understanding of biological phenomena, enabled by rigorous hypothesis-driven approaches imported from physics and formal mathematical logic. These departed from mere trial-and-error approaches that prevailed in previous stages and have enabled all-rational understanding of many key biological processes on the same principles that govern the rest of the material world. This is, after all, the ultimate mission of science as a human endeavour: rational understanding of reality with universal laws and principles.

Yet, the notion that by knowing the functioning of specific biological components we can understand the functioning of whole live systems became insufficient in view of the avalanche of data later generated by the plethora of “omics” technologies. Molecular biology reduces the complexity of a given phenomenon to a point where rigorous logic can be applied, experimental results unambiguously interpreted, and conclusions fixed as permanent pieces of knowledge. Yet, for this to happen, there should be a limited number of actors in an experiment. It thus follows that molecular biology is not capable of handling systems with too many components. Another conceptual and technical framework was clearly required. And thus systems biology [1], which largely relies on network theory (ultimately a branch of physics [2]), came about with the motto that “for understanding the whole, one has to study the whole”.

Using a systems biology approach, motifs, patterns, and correlations can be identified that in turn raise questions and testable hypothesis. However, the scientific agenda up to this point remained mechanistic comprehension of biological phenomena from first principles. The merging of molecular biology with systems biology has produced, for example, a comprehensive theory of gene expression regulation that captures the response mechanisms of live systems to changing scenarios in space and time in a simplified manner. This understanding has enabled the development of many heterologous expression systems and transcriptional circuits that can be parametrised and connected among them, and their behaviour predicted with high accuracy. Adding complexity, regulatory devices can be abstracted as connectable Boolean gates, thereby paving the way to biological computation based on logic circuits, not unlike their silicon-based counterparts [3]. Alas, the underlying assumption that biological systems can be understood as finite state machines (as typical computers are) breaks down when we face biological questions involving a very high number of variables that themselves vary on the fly and thus scape rigorous computation or simple relational logic [4].

There are at least 3 intertwined qualities of life that can make full comprehension of living systems ultimately unreachable. First, they grow, mutate, and evolve. This means that one live object we inspect at a given moment in space and time will not be identical to the same a moment later, let alone if we perturb it to sample or measure specific properties. Second, parameters associated to specific biological devices are context-dependent. One promoter in a location of the genome will show different input–output transfer functions when placed on another site or when cells are grown under different nutritional or environmental conditions. Since the number of such possible conditions is virtually infinite, so is the variability of the parameters at stake. Finally, biological matter is often soft, i.e., not hard in a physical sense. Instead, biology is about flexible materials, plastic shapes, glues, etc. Such components also change their form, which adds another degree of difficulty to making predictions with an accuracy remotely comparable to other branches of science and technology. This does not mean that Biology escapes the laws of physics, but that its ever-growing complexity makes its complete comprehension with standard approaches unreachable—even theoretically. This is the point where machine learning and artificial intelligence (ML/AI) come to the rescue.

ML is about adopting learning algorithms whose objective is to obtain a result (e.g., patterns, rules, correlations) dependent on the input variables (data) but without assuming any prefixed interplay among them. Through the use of computational and statistical methods, such algorithms are trained to make classifications and scores with an explicit degree of reliability. On this basis, it cannot come as a surprise that the large volume of data generated by omics technologies become an excellent input for training ML platforms. In turn, they can deliver dependable predictions on specific, yet complex, biological questions that are not yet amenable to mechanistic understanding.

One revealing case is the prediction of protein folding, a long-standing issue in biology that is now solved to a large extent by the ML/AI-run AlphaFold (AF) [5] and other AI-driven platforms. AF predictions, accurate as they may be, are not built on first principles but largely out of experience and statistical correlations among diverse data on the matter. AF is, in turn, the basis of a whole collection of remarkable platforms for protein engineering, including altogether new-to-nature structures and activities (see, e.g., https://loschmidt.chemi.muni.cz/portal/). To an extent, ML/AI is a return to the experience-based, trial-and-error, black-box approach, which was the source of knowledge in the prescientific era: We know how things behave and how to make them work, but we do not know ultimately why. But … does it matter? **(Box 1).**

**Box 1. A biological principle of uncertainty?**

Mechanistic understanding from first principles anticipates solar eclipses and the successful test of the first prototype of an atomic bomb with 100% accuracy. This is hardly attainable in Biology if the phenomenon at stake has many components. On the other hand, ML-based Alpha Fold enables prediction of complex protein structures with an average accuracy of >80%. True, it is not 100% but it is good enough for moving ahead in most cases. If we accept that we cannot follow all variables of a system—in particular if they change through time and space—resorting to patterns and probabilities might enable us to nevertheless solve practical problems without knowing the underlying basis.

This raises a deeper question about how we study the composition and evolution of biological systems. As physicists gave up on describing the exact position of particles beyond a certain subatomic scale, biologists may also have to give up mechanistic understanding of live systems beyond a given level of complexity in favour of a probabilistic, experience-based approach. In specific scales, mechanistic comprehension—and thus complete predictability—could be inherently impossible. Thus, efforts in that direction are likely to be ultimately futile. Yet, we can still handle description and even understanding of biological systems in terms of chances, scores, and so on.

This should not lead us to disappointment, but rather should be celebrated, as it may open new conceptual frameworks for the understanding of biological phenomena as open-ended occurrences [6] that evolve through trial-and-error progressions, in a way not dissimilar to the processes that ML/AI tries to somehow capture.

Things in biology can become way more complicated than protein folding. Let us take a quite extreme example: the gut microbiome. We know it is an essential partner of the human body that interplays with the immune system, determining health and even psychological well-being through the gut–brain axis. The microbiome encompasses a hypercomplex association of microorganisms of all types undergoing continuous genetic exchange as well as massive molecular trade with the human host. Numerous microbiomes of individuals of diverse age, lifestyles, diets, locations, etc. have been characterised in detail in the last decades, associating distinct patterns of species to either healthy conditions or disease [7] (what has been called dysbiosis). But the enormity of the factors at play makes mechanistic studies virtually impossible. Is that a problem? It could well happen that AI-enabled developments will allow, e.g., for translational development of microbiome science without a deep understanding of how or why. Same in other areas of biology or biotechnology where the need of solutions is more pressing than the importance to understand their basis.

The emphasis on pursuing the laws for understanding the material world could be seen as one of the noblest human undertakings, but it has always been accompanied by the expectation of enabling accurate predictions for practical purposes. The more predictive a branch of science is, the more credible and appealing it is. Biology can make good predictions in systems or subsystems with a limited level of complexity, beyond which it relies on experience-based probabilities. Whether the results of ML/AI are just information or authentic scientific knowledge, as Sydney Brenner would put it [8], remains a legitimate question. A second question is whether there will be a decreasing interest in mechanistic understanding of biological phenomena in favour of using AI to obtain useful rules to make things happen in living systems with a high probability, even if we do not know why they do. Remarkably, even synthetic biology, which has the explicit aim of rational engineering of biology, increasingly relies on ML for adjusting parameters [9], dealing with context sensitivity [10], and guiding the artificial assembly of complex pathways [11]. We should celebrate this latest intersection of biology with a somewhat alien discipline—surely to be followed by other creative encounters in the future.
